# COVID-19: a probable role of the anticoagulant Protein S in managing COVID-19-associated coagulopathy

**DOI:** 10.18632/aging.103869

**Published:** 2020-08-19

**Authors:** Sabyasachi Chatterjee, Tanusree Sengupta, Samarpan Majumder, Rinku Majumder

**Affiliations:** 1 Department of Biochemistry and Molecular Biology, LSU Health Science Center, New Orleans, LA 70112, USA; 2 Department of Chemistry, Sri Sivasubramaniya Nadar College of Engineering, Tamilnadu, India; 3 Department of Genetics, LSU Health Science Center, New Orleans, LA 70112, USA

**Keywords:** Protein S, hypoxia, cytokine storm, IL6, coagulopathy, ACE2, COVID-19

## Abstract

The COVID-19 pandemic has caused monumental mortality, and there are still no adequate therapies. Most severely ill COVID-19 patients manifest a hyperactivated immune response, instigated by interleukin 6 (IL6) that triggers a so called “cytokine storm” and coagulopathy. Hypoxia is also associated with COVID-19. So far overlooked is the fact that both IL6 and hypoxia depress the abundance of a key anticoagulant, Protein S. We speculate that the IL6-driven cytokine explosion plus hypoxemia causes a severe drop in Protein S level that exacerbates the thrombotic risk in COVID-19 patients. Here we highlight a mechanism by which the IL6-hypoxia curse causes a deadly hypercoagulable state in COVID-19 patients, and we suggest a path to therapy.

## INTRODUCTION

The menacing SARS-CoV2 virus has caused a pandemic with over 6.9 million cases and around 400,000 deaths. As of to date (07/11/2020), there are more than 3.2 million confirmed cases and ~134,729 deaths in the U.S. Clinical features of patients admitted to the hospital with the viral disease COVID-19 are bilateral pneumonia, systemic inflammation, endothelial dysfunction, coagulation activation, acute respiratory distress syndrome, and multi-organ failure. Signs of myocardial injury are also observed in at least one quarter of severe cases. Although the lung is the main target organ, the virus can infect other tissues (small intestine, testis, kidneys, heart, thyroid, adipose tissue, colon, liver, bladder, adrenal gland [1]).

The infection risk of SARS-CoV2 has no remarkable correlation with age because the expression of the virus receptor ACE2 does not vary much between young and old; however, mortality is significantly higher in older people compared with the young, (Table 1). SARS-CoV2 infection was found to reduce the expression of ACE2 in lungs, leading to a renin-angiotensin system (RAS) dysfunction. This RAS dysfunction, in turn, would enhance inflammation and vascular permeability in the airways [2].

**Table 1:** Different parameters of COVID-19 patients.

**Place**	**Time and Date**	**No of patient**	**Sex**		**Age**	**Mortality**
			**Male**	**Female**		
Netherlands[42]	7^th^ March - 5^th^ April, 2020	184	139	45	Average : 64	23
Lombardy region of Italy[43]	20^th^ February -18^th^ March, 2020	1591	1304	287	Median : 63	405
Italy[44]	Until 15^th^ March, 2020	22512	13462	9050	Median : 64	1625
Zhongnan Hospital of Wuhan University in Wuhan, China[4]	1^st^ January to 13^th^ March, 2020	449	268	181	Average : 65.1	134
Fatal Cases of COVID-19 from Wuhan China[20]	9^th^ January-15^th^ February, 2020	85	62	23	Median: 65.8	All
Wuhan Jin Yin-tan Hospital, Wuhan, China[45]	Late December, 2019-26^th^ January, 2020	52	36	17	Average: 59.7	32

However, unlike the SARS-CoV pandemic in 2003, COVID-19 is not simply a disease of the upper respiratory tract. COVID-19 patients experience hypercoagulability and increased risk of venous thromboembolism (Table 2). These thrombotic complications have been referred to as *thrombo-inflammation* or *COVID-19-associated coagulopathy* [3–7]. Moreover, several reports indicate that hypercoagulability, as measured by the D-Dimer levels, is present mostly in critically ill and deceased patients (Table 2). In addition to blood clots of all sizes throughout the body, doctors who treat coronavirus patients report a range of other odd and frightening syndromes, such as kidney failure, cardiac inflammation, and immune complications. These syndromes appear to arise from a SARS-CoV2 virus-induced local inflammatory response.

**Table 2:** Studies which indicate that hypercoagulability (supra-physiological levels of D-dimer), is almost always associated with disease severity and mortality of COVID-19.

**Study**	**Sample size**	**Mean D-dimer (<0.5 μg/ml)**	**p-values**	**Comment**
Tang et al, Feb 2020,[34]	Survivors (162)	0.6	<0.001	Disseminated intravascular coagulation (DIC) was found in most deaths
	Non-survivors (21)	2.12		
Han et al, Mar 2020, [33]	Ordinary patient (49)	2.14 ±2.88	<0.001	Huge increase in D-dimer in critically ill COVID patients
	Critical (10)	20.04 ± 32.39	<0.05	
Wang et al, Mar 2020, [46]	ICU (36)	4.14	<0.001	In the non-survivors, D-dimer increased continuously
	Non-ICU (102)	1.66		
Zhang et al, April 2020, [47]	Ordinary (276)	0.41	<0.001	12 non-survivors had D-dimer values greater than 2.0
	Severe (67)	4.76		
Spiezia et al, April 2020, [48]	ICU (22)	5.343 ±2.099	<0.0001	All ICU patients with acute respiratory failure showed severe hypercoagulability, one patient with the most hypercoagulable state died.
Ranucci et al, April 2020, [35]	Total (16)	3.5	0.017	Seven patients died of hypoxia and multi-organ failure
Tang et al, May 2020, [49]	Survivors (315)	1.47	<0.001	30 of the non survivors died even after treated with low molecular weight heparin
	Non-survivors (134)	4.7		

Because of lung involvement, most COVID-19 patients have exceedingly low blood oxygen levels, but, inexplicably, some of these hypoxic patients hardly gasp for breath. Alarmingly, these individuals are subject, without warning, to sudden shortness of breath and massive pulmonary embolism [8]. Note that bleeding is rare in the current onset of the disease.

Our past studies [9–13] with the natural anticoagulant Protein S illuminated our understanding about the significance of Protein S -Factor IXa interaction in hemostasis. Further, we identified a critical role of Protein S in regulating hypoxia and associated thrombotic complications [9].

The overarching goal of this article is to propose a strategy to better control the hypoxemia associated hypercoagulability in severe COVID-19 patients.

### Inflammation, coagulation and hypoxia

Inflammation, as a part of the innate immunity response to an infection, triggers activation of coagulation pathways. Activation of coagulation influenced by inflammation, in turn, can modulate the inflammatory response. The coordinated activation of both coagulation and inflammation during a severe infection is a well-recognized phenomenon known as thrombo-inflammation. Thrombo-inflammation is associated with microvascular thrombosis, hypoxemic respiratory failure, and, in extreme cases, it may lead to death due to development of multiple organ dysfunction syndrome (MODS) [14]. A disproportionate inflammatory response to SARS-CoV2 is associated with exorbitant circulating levels of inflammatory cytokines, which is thought to be a major cause of disease severity and death [15].

The main mediators of inflammation-activated coagulation are the pro-inflammatory cytokines [16]. In severe sepsis, the pro-inflammatory cytokines stimulate mononuclear cells expressing more and more tissue factor that initiate the coagulation pathways. Interleukin 6 (IL-6) is the most important cytokine that influences the expression of tissue factor which activates coagulation.

Thrombo-inflammation – occurs by overproduction of early response proinflammatory cytokines (TNFα, IL-6, and IL-1β) that create a “cytokine storm” [4, 15, 17–22]. This cytokine explosion leads to increased risk of vascular hyperpermeability, multi-organ failure, and eventually death when high cytokine concentrations persist [23, 24]. The inflammatory effects of cytokines also activate vascular endothelial cells and cause endothelial injury with resultant prothrombotic properties [25]. Independent transcriptome datasets from infection models revealed that IL6 is the major cytokine differentially expressed after infection with SARS- CoV2 [26–30].

Autopsies revealed microthrombi in lungs and other organs with associated foci of hemorrhage [31]. Such observations suggest that severe endothelial dysfunction, driven by the cytokine storm and associated hypoxemia, lead to disseminated intravascular coagulation and thromboembolic complications. Importantly, development of local hypoxia will progressively intensify endothelial cell disruption, tissue factor expression, and activation of the coagulation cascade, thereby establishing a deadly positive thrombo-inflammatory feedback loop with thrombosis and hemorrhage occurring in the small vessels of the lungs.

In summary, severe hypoxia is now considered associated with gravely ill COVID-19 patients, and IL6 is upregulated in COVID-19 and promotes *cis* and *trans* signaling to produce a cytokine storm [32]. Further, it is reasonable to conclude that subtle clotting begins early in the lungs, perhaps due to an inflammatory reaction in their fine web of blood vessels, which then sets off a cascade of proteins that prompt blood to clot and prevent proper oxygenation. Blood clots are clearly a major contributor to COVID-19 disease severity and mortality.

### Crosstalk between thrombotic complications and inflammation/cytokine storm in SARS-CoV2 infection

Severity of COVID-19 is commonly associated with coagulopathy; disseminated intravascular coagulation (DIC) being the predominant condition along with high venous thromboembolism rates, and pulmonary congestion with microvascular thrombosis [33]. In general, hemostatic system alterations were indicated by prolonged aPTT, elevated platelet count, increased D-dimer level and fibrin degradation product for patients with severe COVID 19 [34]. Fibrin deposition in alveolar and interstitial lung spaces, in addition to microcirculation thrombosis, may exacerbate respiratory symptoms that require prolonged mechanical ventilation, and which are associated with poor prognosis and death. D-dimer levels have been identified as markers of severity of the disease and predictive of mortality [34]. Ranucci et. al. [35] incorporated viscoelastic tests for ICU patients along with the other commonly performed examinations. The test provides information about clot time (CT), clot strength (CS), fibrinogen contribution (FCS), and platelet contribution (PCS) to clot strength. Patient procoagulant profiles were confirmed by increased CS, FCS and PCS. Increased clot strength has been correlated to high fibrinogen level and somewhat to elevated platelet count.

COVID-19 disease severity is also associated with acute lung injury and hypoxemic respiratory failure, the most common cause of death. High levels of cytokines and chemokines associated with T cell depletion, pulmonary inflammation, and extensive lung damage have been documented in individuals who experienced similar viral respiratory diseases such as SARS and MERS. Thus, the wide-spread lung damage associated with this kind of infection may be caused more by an exaggerated immune response than by the virus itself. In addition, supraphysiological levels of IL-6, IL-10 and TNF-α have been found in the sera of severely ill COVID 19 patients [35]. Therefore, all patients with severe COVID-19 should be screened for excessive inflammation by measuring cytokine levels to stratify patients eligible for a specific immunosuppressive treatment [36].

The prevalence of both a cytokine storm and derangement of coagulation in critically ill COVID-19 patients signifies the aforesaid synergy between inflammation and coagulation. A clear association between increased IL-6 and fibrinogen level was reported for a set of COVID 19 ICU patients [35]. Recent guidance from the International Society on Thrombosis and Hemostasis (ISTH) stresses the need for monitoring coagulation parameters for patients who develop sepsis from the infection. The only widely available standard of care in this respect is a prophylactic dose of low molecular weight heparin, which should be considered for all COVID 19 patients (including non-critically ill) with high D-dimer levels, except for patients in whom anticoagulants are not advisable. For patients allergic to heparin, fondaparinux, a synthetic pentasaccharide, is an alternative. Fondaparinux has antithrombotic activity due to anti-thrombin-mediated selective inhibition of FXa. Systematic anticoagulation therapy for hospitalized COVID 19 patients is now routine treatment.

### IL6, Hypoxia and Protein S

**Figure 1:**
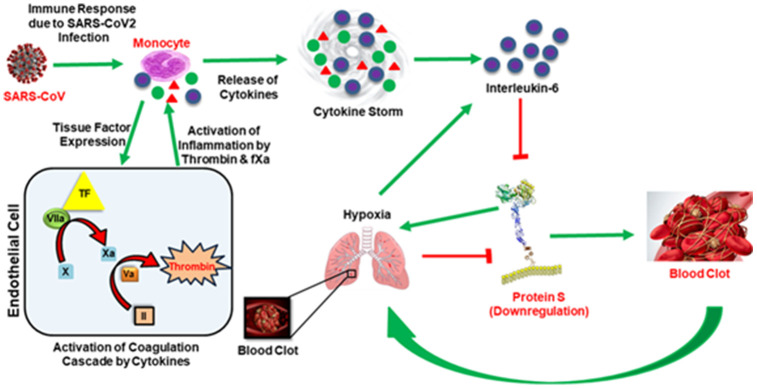
**In the presence of the SAR-COV2 virus, early response proinflammatory cytokines (IL-6, TNFα, IL-1β etc.) are induced and activate the coagulation cascade by stimulating tissue factor (TF) expression from monocytes.** The presentation of tissue factor leads to the formation of thrombin by the TF-VIIa pathway. Thrombin produces clots, and clots get wedged into arteries in the lungs and cause thrombotic complications and hypoxia. Hypoxia also induces IL-6. Simultaneously, thrombin augments inflammation and accelerates the production of proinflammatory cytokines, termed ‘cytokine storm’. Both cytokine storm and hypoxia downregulate Protein S, leading to coagulopathy. Green arrows represent upregulation and red blockage represent downregulation.

An overlooked aspect of hypoxia and the IL6-induced cytokine storm is that *both* factors downregulate a key anticoagulant, Protein S [9, 32] (Figure 1). For example, in a population of stroke patients, IL6 was upregulated, and it caused downregulation of Protein S that resulted in venous thrombosis [37]. We demonstrated that hypoxia downregulates Protein S expression in HepG2 cells [9]. Further, we showed that Protein S supplementation in thrombotic mice (mimicking hypoxic niche due to constitutive stabilization of HIF1α) plasma was able to alleviate the thrombotic risk [9]. Notably, addition of Protein S in normal mice plasma reduced thrombin generation as well [9]. These data indicate that Protein S supplementation could be useful in treating thrombotic complications. A substantial number of severe COVID-19 patients manifest both hypoxia and prothrombotic complications [34, 38–40] and we speculate that reduced Protein S level might play a key role in the disease progression of these patients.

Ordinarily, Protein S deficiency is due either to homozygous or heterozygous genetic alteration, and Protein S deficiency can result from various pathological states and diseases. In all cases, Protein S deficiency is associated with a higher risk of venous thrombosis. Because both hypoxia and IL6-induced inflammation depress Protein S abundance, it’s reasonable to consider administration of Protein S as an effective therapy in severe Covid19 patients. Indeed, therapeutic heparin has improved the conditions of COVID-19 patients who experienced hypoxia. However, heparin targets FIXa, FXa and thrombin [41] through antithrombin. Therefore, direct administration of Protein S should have a highly specific anticoagulant effect in any thrombotic complications caused by Protein S deficiency. Of course, the possibility of bleeding would need attention, but, fortunately, even high doses of heparin have not caused bleeding in COVID-19 patients. Nonetheless, before Protein S administration can be deemed a new therapeutic approach, it is necessary to determine the extent to which Protein S is downregulated in a large cohort of COVID-19 patients. In view of the double curse of hypoxia and IL6, we expect Protein S deficiency to be severe in COVID-patients. However, we acknowledge that testing for safety and efficacy as well as FDA approval would be required before this approach could be implemented.
